# Valorisation to biogas of macroalgal waste streams: a circular approach to bioproducts and bioenergy in Ireland

**DOI:** 10.1007/s11696-016-0005-7

**Published:** 2016-12-16

**Authors:** Silvia Tedesco, Joseph Stokes

**Affiliations:** 1 Faculty of Science and Engineering, School of Mechanical Engineering, Manchester Metropolitan University, Dalton Building, Chester Street, Manchester, M1 5GD UK; 2 Department of Mechanical and Manufacturing Engineering, Dublin City University, Glasnevin, Dublin, 9 Ireland

**Keywords:** Integrated biorefinery, Macroalgae, Methane, Biogas, Anaerobic digestion, Algal residues

## Abstract

Seaweeds (macroalgae) have been recently attracting more and more interest as a third generation feedstock for bioenergy and biofuels. However, several barriers impede the deployment of competitive seaweed-based energy. The high cost associated to seaweed farming and harvesting, as well as their seasonal availability and biochemical composition currently make macroalgae exploitation too expensive for energy production only. Recent studies have indicated a possible solution to aforementioned challenges may lay in seaweed integrated biorefinery, in which a bioenergy and/or biofuel production step ends an extractions cascade of high-value bioproducts. This results in the double benefit of producing renewable energy while adopting a zero waste approach, as fostered by recent EU societal challenges within the context of the Circular Economy development. This study investigates the biogas potential of residues from six indigenous Irish seaweed species while discussing related issues experienced during fermentation. It was found that *Laminaria* and *Fucus* spp. are the most promising seaweed species for biogas production following biorefinery extractions producing 187–195 mL CH_4_ gVS^−1^ and about 100 mL CH_4_ gVS^−1^ , respectively, exhibiting overall actual yields close to raw un-extracted seaweed.

## Introduction

The world seaweed industry is estimated to be worth US$5.5–6 billion annually, with US$ 5 billion being generated from products destined for human consumption (Roesijadi et al. 2010; Walsh and Watson 2011). Currently, seaweeds are used not just for human food, but in a variety of advanced applications. A wide range of food supplements, fertilizers, cosmetics and pharmaceuticals are now produced from seaweeds, and these specialisations hold the greatest opportunity for those involved in the seaweed processing industry in Ireland. Ireland’s vast marine resources account for ten times the land area. Ireland has an estimated national seaweed harvest of 25,400 fresh tonnes per annum, 100% of which is wild (Walsh and Watson 2011). However, it has been estimated that Ireland has at least 3 million tonnes of standing kelp (Bruton et al. 2009), which is not being exploited.

Anaerobic digestion of Irish seaweed to biogas has been investigated by several scientists (Tedesco et al. 2013, 2014a, b; Vanegas and Bartlett 2013a, b), who reported methane yields similar to those from the most promising land-based energy crops. In general, however, seaweed-based biogas is not consistently economically viable due to the cost of the stock (Roesijadi et al. 2010; van Hal et al. 2014), and its fluctuating intrinsic biochemical properties and other technological bottlenecks (Montingelli et al. 2015; Roberts and Upham 2012), which make its use (Li et al. 2013) too expensive for energy production purposes only.

According to the Sea Change Strategy (2006), and confirmed by the Irish Fishery Board (BIM), the Irish seaweed production and processing industry will be worth €30 million per annum by 2020 (Strategy 2007). In Ireland, the main seaweed bioproducts currently produced consist of: animal nutrition, animal hygiene, plant health, soil fertilizers, alginate, cosmetics and nutraceutical products (Irish Macroalgae Industry 2011). When processed for high-value compounds extraction, a significant amount of sugar-rich seaweed residues is generated and needs to be disposed of, creating an opportunity for a biofuel/bioenergy production step by following an integrated biorefinery approach. Biorefineries with integrated biomass conversion processes can produce fuels, electricity and heat along with valuable chemicals. The implementation of an integrated biorefinery approach is believed to help make seaweed exploitation to bioenergy economically feasible (Burton et al. 2009; Hughes et al. 2012).

Recent studies suggest that macroalgae have high potential as feedstock for biorefinery to produce biomaterials and bioenergy (Hughes et al. 2012; Jung et al. 2013), while biogas production from macroalgae was found to be more technically viable than for other biofuels (Roesijadi et al. 2010; Jung et al. 2013). Furthermore, the feasibility of fermenting waste solids and liquids from seaweed processing plants to generate biogas on site is a research priority according to Sustainable Energy Authority of Ireland (SEAI) (Burton et al. 2009). However, it is not known how the high-value bioproducts extraction will affect biogas production from seaweed residues. It has been estimated that for example the extraction of alginate, laminaran and fucoidan would lower by almost 50% the amount of fermentable sugars from brown seaweed (Burton et al. 2009; van den Burg et al. 2013). Biogas yields from *Ascophyllum* spp. residues from alginate extraction in Scotland were found to range between 376 and 360 NL kgVS^−1^ with methane content between 62 and 63% depending on digesting temperature and digester configuration in batch mode (Edyvean 1988). In Norway a mix of alginate extracted residues from *Laminaria hyperborea* and *Ascophyllum nodosum* yielded 100–150 NL kgVS^−1^ in batch mode depending on the same conditions (Kerner et al. 1991). Both of the above studies show that seaweed biorefinery with integrated biogas production is convenient as the obtained biogas yields compare favorably with other substrates.

The proposed work aims to assess the biogas potential of waste seaweed residues downstream of existing industrial extraction processes of high-value products from the Irish macroalgae processing industry. Therefore, this study aimed to characterize the feedstock’s biochemical composition and lead to: (1) the identification of potential methane yields, (2) the most promising seaweed species of six among *A*. *nodosum*, *Laminaria digitata* and *hyperborea*, *Fucus serratus* and *vesiculosus*, and *Ulva rigida*, (3) understand how bioproducts extraction affects composition, and thus methane yield of the seaweed residues.

## Experimental

### Materials and methods

Biomass residues of *F*. *serratus* (FS), *F*. *vesiculosus* (FV), *A*. *nodosum* (AN), *L*. *digitata* (LD), *L*. *hyperborea* (LH) and *U*. *rigida* (UR) were collected in October after extraction of high value compounds at laboratory scale performed by an Irish seaweed company in Co. Galway and Co. Clare, Ireland; and then frozen to −20 °C until use. The extracting procedures adopted by the company were targeted to the extraction of alginic acid, fucoidan, fucoxanthin, laminarin, mannitol, and proteins. Dry organic matter or Total Solids (TS) and Volatile Solids (VS) were identified using a high-temperature oven via overnight drying at 105 °C followed by combustion at 575 °C of the seaweed residues, as by standard procedure by (Ehrman 1994). Results of the proximate composition analysis are shown in Table 1, which also includes the findings of the total chemical oxygen demand (tCOD) test. COD is widely used to evaluate the amount of organic matter within water and wastewater. This measurement has been taken to estimate the organic matter dissolved in the samples. The procedure for tCOD analysis was performed as proposed by Hach (1999).

**Table 1:** Dry matter and organic fraction in the seaweed residues

Species	Total solids (TS) (%)	Volatile solids (VS) (%TS)	tCOD (mg L^−1^)
*Fucus serratus* (FS)	27.7	81.0	11,100.0 ± 5
*Fucus vesiculosus* (FV)	34.1	77.7	8333.3 ± 3
*Ascophyllum nodosum* (AN)	32.6	78.8	7033.3 ± 4
*Laminaria digitata* (LD)	22.3	75.0	9400.0 ± 3
*Laminaria hyperborea* (LH)	26.6	84.0	13,233.3 ± 2
*Ulva rigida* (UR)	20.5	73.3	4200.0 ± 4

An ultimate analysis was then conducted to identify the elemental composition of the fermenting substrates. A COSTECH elemental analyser CHNS-O, model 4024 was used to estimate the mass percentages of each element among carbon, hydrogen, nitrogen, sulphur and oxygen. The elemental analyser’s internal configuration needs to be modified for H_2_O absorption when switching from CHNS to O mode to detect the percentage of the oxygen element. Therefore, the ultimate analysis was performed in two steps characterized by different regression factors. The machine was calibrated against a known standard (N = 6.5%, C = 72.5%, H = 6.09%, S = 7.44%, and O = 35.5%), with correlation between 0.982 and 0.999 for the elements CHNS, and 0.993 for O. Triple replicates were used for each unknown sample, and consequently for each seaweed species. Results are shown in Table 2. A biogas analyser, model Drager X-Am 3000, was used to verify anaerobic conditions were created correctly when preparing the reactors and to analyze the gas composition at the end of the gas collection. An upturned measuring cylinder was utilized to derive the biogas volume, respectively, at days 2, 3, 7, 10, 12 and 15 of retention time.

**Table 2:** Ultimate Analysis of the seaweed residues with standard deviation values

spp.	C^a^ (%)	H^a^ (%)	N^a^ (%)	S^a^ (%)	O^a^ (%)	C/N
FS	41.4 ± 0.7	4.9 ± 0.1	1.5 ± 0.1	–	35.1 ± 0.2	27.6
FV	45.1 ± 0.6	5.1 ± 0.2	1.5 ± 0.2	–	36.5 ± 1.2	30.7
AN	46.4 ± 1.9	5.2 ± 0.4	1.5 ± 0.1	0.7 ± 0.3	34.8 ± 1.0	30.3
LD	38.9 ± 0.1	4.7 ± 0.1	1.3 ± 0.1	–	37.2 ± 1.7	30.7
LH	42.0 ± 1.0	5.1 ± 0.2	0.9 ± 0.2	–	39.0 ± 2.2	45.0
UR	40.6 ± 0.2	5.0 ± 0.1	3.6 ± 0.1	1.1 ± 0.2	35.3 ± 0.2	11.3

^a^Molecular weight: C = 12.01, H = 1.01, N = 14.00, S = 32.07, O = 15.99

### Theoretical methane yields and bioreactor preparation

Elemental composition percentages from the ultimate analysis were used to derive the theoretical stoichiometric methane potential (SMP) by means of the well-known stoichiometric Buswell formula in (1) (Symons and Buswell 1933):

1 $${\text{C}}_{\text\it{c}}{\text{H}}_{\text\it{h}} {\text{O}}_{\text\it{o}} {\text{N}}_{\text\it{n}} {\text{S}}_{\text\it{s}} { + 1/4}( 4 {\text\it{c}} - {\text\it{h}} - 2 {\text\it{o + 3n + 2s}}){\text{H}}_{ 2} {\text{O} = 1/8}(4{\text\it{c + h}} - 2 {\text\it{o}} - 3 {\text\it{n}} - 2 {\text\it{s}}){\text{CH}}_{ 4} { + 1/8}(4{\text\it{c}} - {\text\it{h + 2o + 3n + 2s}}){\text{CO}}_{ 2} \;+{\text{\;{\it n}NH}}_{ 3} \;+{\text{\;{\it s}H}}_{ 2} .$$

Measurements of the pH of the samples prior to and at the end of the digestion were taken using a Hanna pH meter, model 213. The initial pH was considerably alkaline for all the seaweed residues as a result of the extraction process adopted. The values of pH ranged between 8.9 and 9.3, falling out of the ideal pH range for anaerobic digestion (AD). pH is a very important factor in AD and one of the key parameters defining the stability of a digester. Ideally the pH suitable for anaerobic digestion of seaweed varies between 7.5 and 8.5 (Kelly and Dworjanyn 2008). Therefore, before the actual fermentation experiment, the pH value of the samples was decreased using a 0.1N sulphuric acid solution while constantly stirring until neutral pH was reached.

Digesting reactors were prepared with 10 g of fresh pH-adjusted residues per species, which were diluted in 100 mL of tap water, and coarsely chopped to roughly 0.5–1 cm particle size. This specific residues-to-water proportion was selected to produce a 1:10 biomass-to-water ratio, for comparison with findings from previous work on milled seaweeds (Tedesco et al. 2013, 2014a, b). To add the necessary fermenting microorganisms to the reactors, the samples were then incubated with 300 g of digested sewage sludge (TS = 4.8%; elemental composition: C = 50.8%, H = 6.3%, N = 2.1%, and O = 35.4%), provided by the wastewater treatment plant of Celtic Anglian Water (CAW) Ltd. The sludge’s pH was measured as 8.1 ± 0.03.

In the previously mentioned works, the inoculation of seaweed reactors with sludge provided by CAW Ltd. resulted to have a self-buffering effect favourable to methane production and a C/N balancing capacity in the reactors. In fact, by adding sludge to the samples in this study in the mass ratio of 3:1, the final C/N ratio resulted in 24 ± 1 in all reactors which is in range with ideal settings, having an initial pH of 7.5–8.0 before digestion. Each reactor condition was reproduced in triplicate. Reactors fermentation was allowed for 15 days, which corresponds to about 75% of the usual digestion time applied to lab-scale seaweed co-digestion in batch mode. Such residence time was selected on the basis of previous experience with digestion of milled seaweed, as more than 80–90% of the yielded biogas is produced within the second week after incubation. The digestion temperature was set at 39 ± 1 °C. The sludge contribution to the biogas formation was 798 mL across the digestion period, 35% of which was methane. Such contribution was subtracted to the co-digestion yields to determine the actual yields of the residues. A biodegradability index (BI) was used to estimate the digestion efficiency and calculated as a % of the SMP yield achieved at the end of the digestion period, refer to Eq. (2):

2 $${\text{BI}} \% = \frac{{{\text{SMP}} - {\text{Actuals}}}}{\text{SMP}} \times 100.$$

## Results and discussion

### Substrate’s composition effect on methane production

The obtained TS values shown in Table 1 indicate that the seaweed residues lost moisture content during the extraction process, due to osmotic gradients of solvents used in the extraction itself and its related chemical reactions. In fact, it is well known that algal biomass exhibits very high levels of moisture content. Water content in seaweeds ranges between 78 and 90% (Burton et al. 2009; Marinho-Soriano et al. 2006) depending on species and period of harvesting, while in this study it was found to be between 65 and 80%, thus resulting in higher TS% w/w compared to fresh biomass. The VS fraction of the residues remained very high (above 70%) for all species despite the extraction processes, with values in line with literature for un-extracted seaweed (Horn 2009), suggesting high biogas potential. Total COD (tCOD) concentrations found in the residues are in line with those obtained by (Nkemka and Murto 2010) from seaweed leachate. However, they are up to twofold below values reported by (Gurung et al. 2012), where raw seaweeds were used. The explanation of this behaviour is the composition of the sample, which may consistently vary across seasons and among seaweed species. Also, sample preparation procedure has a significant effect, i.e. organic solids concentration in the batch reactors can be selected arbitrarily for testing or be based on previous experience. In fact, sample settings of this study are closer to trials set up by (Nkemka et al. 2010), in terms of solids concentration and biomass harvesting period (September). This result is encouraging, as tCOD values from raw seaweed are in range with those from the residues under investigation, confirming that a valuable amount of organic matter is present.

Methane production is known to be positively correlated to the contents of carbon and hydrogen, while being negatively related to the oxygen content. The high percentages of hydrogen and sulphur (Table 2) already indicate that formation of corrosive nitrogen and sulphur containing emissions is likely to occur. In Table 2, the content of carbon and hydrogen suggests methane yields close to those obtainable from starch (415 mL gVS^−1^) (Angelidaki and Ellegaard 2004). High content of nitrogen and sulphur may also lead to inhibition of the methanogenic phase, besides forming toxic gases such as ammonia and hydrogen sulfide.

C/N ratios ranging from 20 to 30 are considered optimal for AD, as if this ratio is very low nitrogen will be released and accumulated in the form of ammonium ion (NH_4_^+^) (Chandra et al. 2011). As it can be observed in Table 2, residues of FS, FV, AN, LD are perfectly in range of the ideal conditions, while digesters containing LH and UR residues should be complemented with another waste to balance the C/N ratio. Nitrogen rich and carbon rich substrates should be, respectively added to the seaweed digester containing LH and UR.

### Theoretical and effective methane yields

Results of the fermentation experiment are reported in Table 3. Tedesco et al. (Tedesco et al. 2013, 2014a, b; Vanegas and Bartlett 2013a, b) reported biogas and methane yields from *L*. *digitata* and *hyperborea*, *Ulva* and *Fucus* species in Ireland. The mentioned studies will serve as a comparison to the biogas yields obtained in this investigation, as they were conducted on fresh un-extracted seaweed biomass.

**Table 3:** Biogas yields and composition in the actual fermentation of seaweed residues

Species	Biogas produced (mL gVS^−1^)	Methane content (%)	NH_3_ (ppm)	H_2_S (ppm)
FS	252.9	40	>300	50–70
FV	223.9	46	70	>100
AN	195.7	43	>300	40
LD	425.5	44	60–100	>100
LH	453.7	43	55	>100
UR	182.8	40	20	>100

Biogas yields obtained from *Fucus* spp. residues (about 100 mL CH_4_ gVS^−1^) were found slightly above the highest values obtained by (Tedesco et al. 2013) conducted for 21 days in which the inoculum-to-substrate ratio (ISR) was 1:1 w/w and the biomass-to-water mass ratio was 1:20 w/w. The amount of TS in the reactors was thus doubled in this study’s fermentation, while the volume of inoculum was instead one third of that used in this experiment. Beside the different ISR settings between (Tedesco et al. 2013) and this study, seasonal variation in biochemical composition has also an influence, as methane yields are meant to be higher in warm seasons and lower in the cold ones. This is due to higher carbohydrates stored by the plant during spring and summer. Biomass composition being equal, the larger the initial amount of inoculum in the reactors and the faster the digestion occurs. In general, considering the different ISRs and biochemical seasonal variation, these studies would suggest that *Fucus* spp. residues should maintain their biogas yield around 200–230 mL gVS^−1^ for digestion at mesophilic temperatures throughout the year.

Biogas yields from *L*. *digitata* and *hyperborea* are in line with average biogas and methane yields obtained by Tedesco et al. (Tedesco et al. 2014a, b). Lower yields were obtained by (Vanegas and Bartlett 2013a, b) for LD, FS and *Ulva* spp. than those achieved in this study. However, this could be attributed to the use of bovine slurry as inoculum, which creates an increasingly acid environment for the bacteria due to accumulation of volatile fatty acids (VFAs) in the bioreactors. In fact, Vanegas et al. had to adjust the pH within the reactors by adding a mixture of NaOH and 10% KHCO_3_ to the reactors to re-establish the ideal pH range and boost the buffer capacity of the system. It should be pointed out that previous investigations carried out by the authors and the literature (Chynoweth et al. 1981; Hanssen et al. 1987) found the use of sludge as inoculum to actively contribute to the digestion stability and contrast VFAs accumulation followed pH decrease, as this has happened frequently when digesting seaweeds. It can be concluded that sludge should be considered as first inoculum/co-substrate option when planning a seaweed digesting facility.

Finally, previous unpublished work from the authors identified a biogas yield of 336 mL gVS^−1^ from *Ascophyllum nodosum* (March), while Hanssen et al. (Hanssen et al. 1987) found a yield of 280 mL gVS^−1^ (September). These values are 30–42% higher than those from the AN residues from this study. Furthermore, research conducted in Scotland (Edyvean et al. 1988) reported biogas yields up to 376–360 mL gVS^−1^ from AN residues after alginate extraction only. The low biogas yield obtained from AN residues in this investigation can be attributed to some extent to the multiple extractions performed, which are not limited to alginic acid, and are, therefore conceptually coherent. The biochemical composition of AN has been found by the literature to be much less affected by seasonal variation compared to most brown seaweeds (Black 1948). Biogas and methane yields from AN could so be considered rather stable during the year and relied upon, other conditions being equal. However, this is currently being experimentally verified for AN residues following biorefinery extractions in a broader investigation.

Interestingly, the overall methane content (Table 3) in the biogas that ranged between 40 and 45% for all species. Such a small variation is certainly connected to the fact that the C/N ratio was set to the same value in all reactors. This highlights the importance and the role played by this parameter in the methanation process. Values of NH_3_ and H_2_S were very high and even higher values are envisaged at plant scale. This would either slow down or prevent methane formation, as excessive NH_3_ and H_2_S concentrations are toxic to methanogens. Furthermore, these organic acid gases are corrosive to pipes and engines, as well as dangerous to human health in the detected concentrations. Consequently, the biogas obtained should be cleared up before use in combined heat and power (CHP) units or their formation should be chemically prevented via the addition of iron-based chemicals to the digester (Streefland et al. 2010).

**Fig. 1:**
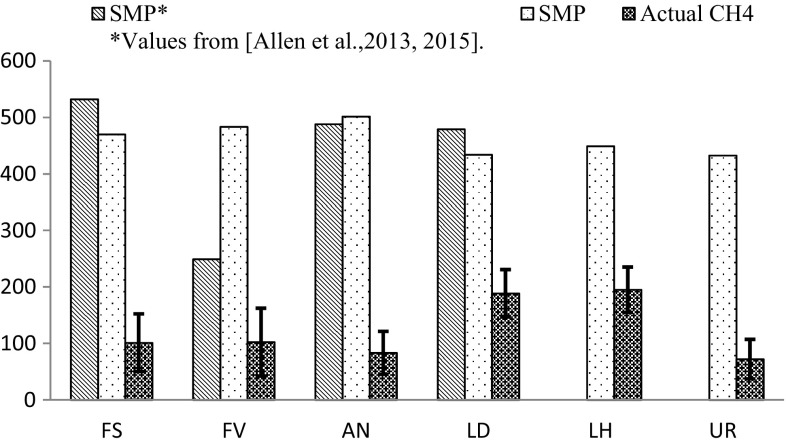
Stoichiometric (SMP) and actual methane yields (mL gVS^−1^)

**Fig. 2:**
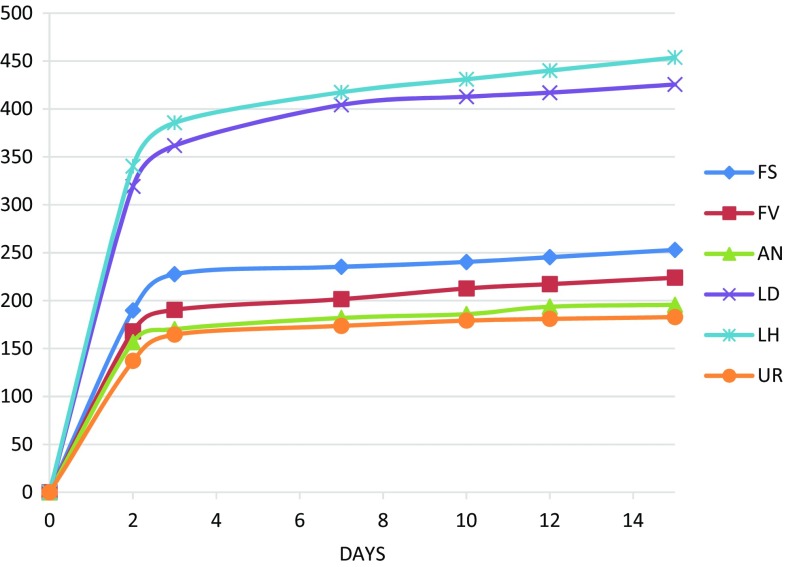
Cumulative biogas yields across 15 day digestion (mL gVS^−1^)

The SMP and actual methane yields from the analysed residues were compared with theoretical yields from existing literature in Ireland (SPM*) from un-extracted seaweed in Fig. 1, while Fig. 2 shows the cumulative biogas yields achieved across the digestion period. It can be noticed that the derived SPM yields are close to those theoretically achievable from most anaerobically digested lignocelluloses such as maize (420 mL gVS^−1^) and Miscanthus (488 mL gVS^−1^), as reported by (Lübken et al. 2010). Furthermore, most theoretical yields are in line with those achieved by a very recent study conducted in Ireland during the summer 2013 on the coasts of Cork (Allen et al. 2015). However, practical yields are considerably below the theoretical maximum as only a fraction of VS is normally destroyed and transformed into methane by fermenting microorganisms. The volatile solids reduction is generally associated to the recalcitrance of the substrate to biological degradation, as it clearly shows in Fig. 2. In this study, however, such a result also partially reflects the short digestion time used, i.e. 15 days.

Actual methane yields from AN and UR residues were the lowest obtained. AN is known to contain polyphenols which are difficult to degrade and can inhibit anaerobic digestion. Hence, AN residues would produce more methane when pre-treated with formaldehyde (Horn 2009; Vanegas and Bartlett 2013a), which would eliminate issues associated with polyphenols poisoning. *Ulva* spp. conversion to methane is believed to have suffered from excessive concentrations of dissolved NH_3_, which developed an inhibition. In fact, UR was the substrate with the highest nitrogen content (Table 2). This is also demonstrated by extremely low gaseous ammonia concentration in the resulting biogas, i.e. 20 ppm, which indicates that most of the nitrogen remained dissolved in the substrate, and had a toxic effect on methanogens. This suggests that other substrates different from sludge and much poorer in nitrogen should be tested for co-digestion with this particular residue, with a C/N above 25. Furthermore, such low methane yields can also be attributed to low tCOD value in the bioreactor.

*Laminaria* spp. residues exhibit the highest potential for methane production, with a yield of 187–195 mL CH_4_ gVS^−1^. However, these substrates realised only 43% of their potential, while an average of about 20% of the theoretical yield was produced by the other species. The actual methane yields shown in Fig. 1 were obtained with no pre-treatment and by fermenting the residues after coarse chopping and initial pH adjustments only. A variety of pre-treatments have been proven to be very effective at enhancing the methane conversion efficiency from algal biomass, but they would increase the operational cost of the anaerobic digestion plant, sometimes making the whole process not economically viable as most pre-treatments are energy intensive or impact the process sustainability due to the use of chemicals (Jard et al. 2013). However, as the structural integrity of the seaweed is preserved after the compounds extraction (residues provided were not reduced in particle size), an initial milling/shredding of the residues will be indispensable to the automation of the process and to maximise the surface contact with bacteria within the digester (Bernat et al. 2015).

AN, *Fucus* spp. and UR residues performed very poorly compared to their methane potential (BI 17–22%). Technically there is a large room for improvement of the BI, and consequently of the methane yields. Previous work (Tedesco et al. 2014a) has indicated that for example, mechanical comminution of the biomass can enhance the methane yields from *Laminaria* spp. up to 53% (290 mL CH_4_ gVS^−1^), and unpublished work indicated that ground AN can reach up to 170 mL CH_4_ gVS^−1^ around the end of August.

*Ulva* spp. are abundant among the so-called ‘drift seaweeds’. These species’ plant structure is very fragile and storms are able to detach them from their roots, often causing green tides in Ireland (Allen et al. 2013). Given the low methane yield of UR residues and the general uncertain availability of the stock whether farmed or wild harvested, it would not be recommendable to rely on this substrate to produce gaseous fuel. *Fucus*, *Laminaria* and *Aschophyllum* spp. are sub-tidal plants more tightly attached to the rocks of the sea floor. If in the future, seaweed will be cultivated in Ireland for food, bioactive compounds and energy extraction, the latter mentioned species (FS, FV, LD, LH and AN) hold the highest potential for all these applications.

## Conclusions

Seaweed integrated biorefinery has substantial unexploited potential in Ireland for production of high-value bioproducts, heat, power and biofuels. In this study seaweed residues were investigated for biogas production following biorefinery extractions. The theoretical methane yields obtained were found comparable to un-extracted seaweeds and to popular land-based crops, even following extraction of bioproducts.

*Laminaria* and *Fucus* species hold the best potential for biogas production. However, all the seaweed residues realised below 50% of their stoichiometric methane potential, thus leaving large room for improvements of the biomass’ biodegradability index. This can be achieved via the use of the most advanced existing pre-treatment technologies, which have been extensively proven by the literature to have a beneficial effect at enhancing performance and shortening digestion time. Nevertheless, it is recommended that cost of pre-treatment is taken into consideration and analysed from a cost-benefit perspective with respect to the extra methane achieved. This study’s results indicate that a seaweed integrated biorefinery approach is possible in Ireland and will benefit existing seaweed bioproducts stakeholders by generating energy from their internal processes’ waste streams.

## Abbreviations

tCOD: Total chemical oxygen demand (mg L^−1^)
SMP: Stoichiometric methane potential (mL gVS^−1^)
TS: Total solids (%)
VS: Volatile solids (%)
